# CPNE7 promotes colorectal tumorigenesis by interacting with NONO to initiate ZFP42 transcription

**DOI:** 10.1038/s41419-024-07288-z

**Published:** 2024-12-18

**Authors:** Liangbo Zhao, Xiao Sun, Chenying Hou, Yanmei Yang, Peiwen Wang, Zhaoyuan Xu, Zhenzhen Chen, Xiangrui Zhang, Guanghua Wu, Hong Chen, Hao Xing, Huimin Xie, Luyun He, Shuiling Jin, Benyu Liu

**Affiliations:** 1 Tianjian Laboratory of Advanced Biomedical Sciences, Academy of Medical Sciences, Zhengzhou University, Zhengzhou, China; 2 Department of Oncology, The First Affiliated Hospital of Zhengzhou University, Zhengzhou, China; 3 First Clinical Medical College, Zhengzhou University, Zhengzhou, China; 4 School of Life Sciences, Zhengzhou University, Zhengzhou, China; 5 Department of Pathophysiology, School of Basic Medical Sciences, Zhengzhou University, Zhengzhou, China; 6 Institute of Infection and Immunity, Henan Academy of Innovations in Medical Science, Zhengzhou, China

**Keywords:** Colon cancer, Mechanisms of disease

## Abstract

Colorectal cancer (CRC) is the third most common cancer worldwide and the second leading cause of cancer-related death globally. Also, there is still a lack of effective therapeutic strategies for CRC patients owing to a poor understanding of its pathogenesis. Here, we analysed differentially expressed genes in CRC and identified CPNE7 as a novel driver of colorectal tumorigenesis. CPNE7 is highly expressed in CRC and negatively correlated with patients’ prognosis. Upregulation of CPNE7 promotes proliferation and metastasis of cancer cells in vitro and in vivo, and vice versa. Mechanistically, CPNE7 interacts with NONO to initiate ZFP42 transcription, thus promoting CRC progression. Moreover, ZFP42 knockdown inhibits tumor cell proliferation and migration while promoting apoptosis. Notably, delivery of CPNE7 shRNA or the small molecule gramicidin, which blocks the interaction between CPNE7 and NONO, hinders tumor growth in vivo. In conclusion, our findings demonstrate that the CPNE7-NONO-ZFP42 axis promotes colorectal tumorigenesis and may be a new potential therapeutic target.

## Introduction

Colorectal cancer (CRC) is the third most common cancer worldwide and the second leading cause of cancer-related deaths globally [1]. It is estimated about 520 thousand new CRC cases and 240 thousand CRC deaths occurred in China in 2022 and the incidence of CRC in China is increasing year by year [2–4]. Current treatments for CRC include surgery, radiotherapy, chemotherapy and immunotherapy [5]. Combining surgical treatment with radiotherapy and chemotherapy can reduce cancer mortality. However, patients are prone to relapse. Besides, some tumors are not suitable for surgery due to their location or patient intolerance [6]. Targeted therapy and immunotherapy are limited to specific CRC patients. For example, EGFR monoclonal antibody such as cetuximab and panitumumab can be used only for patients with wildtype KRAS and RAF, but their efficacy may be compromised by drug resistance and side effects [7]. Therefore, it is critical to investigate the molecular mechanisms of CRC pathogenesis and develop novel potential therapeutic targets.

CPNE7 (copine7), a member of the copine family, locates on human chromosome 16 [8]. CPNE7 contains two domains: the N-terminal plasma membrane-binding domains (C2A and C2B) and the C-terminal protein-binding domain [9]. Current research on CPNE7 mainly focuses on tooth development and differentiation [10, 11]. For instance, CPNE7 secreted from pre-ameloblasts promotes odontoblast differentiation via epithelial-mesenchymal interaction [12]. While CPNE7 silencing inhibits the proliferation and osteogenic differentiation of human periodontal ligament cells [13]. Therefore, CPNE7 derived peptide could be used in tooth caries treatment and dental restoration [14–16]. CPNE7 is also involved in the regulation of some diseases such as nonalcoholic fatty liver disease [17]. Importantly, a recent study indicates CPNE7 may be a tumor driver [18]. Up-regulated CPNE7 activates the NF-κB pathway in mesenchymal stromal cells and promotes metastasis of oral squamous cell carcinoma [18]. However, further investigation is needed to determine how CPNE7 participates in tumorigenesis and whether it can serve as a new therapeutic target.

ZFP42 (zinc finger protein 42), also known as REX1 or ZNF754, is a zinc finger protein [19] located on human chromosome 4. It has two transcripts, both encoding a protein of 310 amino acids. ZFP42 is a transcription factor expressed in the early embryo [20, 21] and it is widely used as a stem cell marker [22, 23]. Emerging studies report that ZFP42 is also expressed in tumor cells [24, 25]. ZFP42 activates the MEK/ERK pathway, promoting tumorigenesis in prostate cancer [25]. In cervical cancer, ZFP42 promotes epithelial-mesenchymal transition (EMT)-induced metastasis by activating the JAK2/STAT3 signaling pathway [24]. In hepatocellular carcinoma, ZFP42 inhibits transcription of MKK6, then reduces p38/MAPK signaling and suppresses hepatocarcinogenesis [26]. However, how ZFP42 regulates the CRC remains unclear. Here, we report that CPNE7 is highly expressed in CRC and predicted poor prognosis. CPNE7 is critical not only for CRC cell proliferation, migration and survival in vitro but also for tumorigenesis and metastasis in vivo. Mechanistically, CPNE7 promotes ZFP42 transcription by interacting with NONO. Furthermore, intratumoral injection of CPNE7 shRNA or gramicidin inhibit CRC progression in vivo. Taken together, our findings demonstrate that CPNE7 is a key gene that promotes the growth and metastasis of CRC, and it may be a strong candidate target for CRC therapy.

## Results

### CPNE7 is upregulated in CRC

**Fig. 1:**
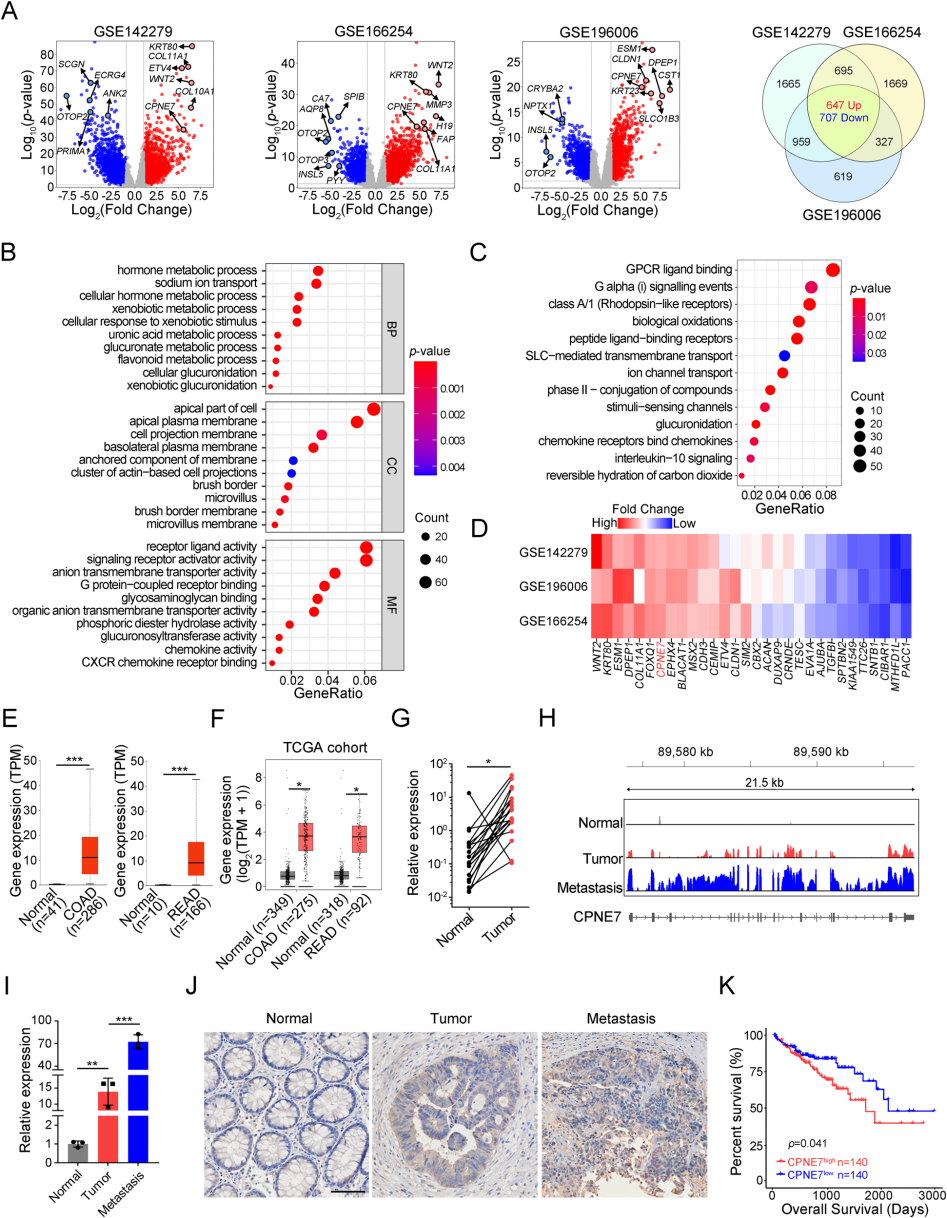
CPNE7 is upregulated in CRC. **A** Volcano plots and Venn plot of DEGs in GEO CRC datasets GSE142279, GSE166254 and GSE196006. The selection criteria for DEGs were *p*-value < 0.05 and |FoldChange | >2. **B** GO analysis of common 1,354 DEGs in (**A**). BP: Biological Process, CC: Cellular Component, MF: Molecular Function. **C** KEGG analysis of common 1,354 DEGs in (**A**). **D** Heat map of the top 30 genes from 647 common upregulated DEGs in (**A**). **E**, **F** CPNE7 expression in COAD, READ and normal tissues in UALCAN database (**E**) and GEPIA database (**F**). TPM: Transcripts Per Million. **G** CPNE7 expression in 19 pairs of CRC samples were detected by qRT-PCR. **H**, **I** *CPNE7* mRNA expression in normal tissues, tumor tissues and liver metastatic tissues measured by RNA-sequencing (**H**) and qRT-PCR (**I**) in paired CRC samples. **J** Immunohistochemical staining of CPNE7 in normal tissues, tumor tissues and liver metastatic tissues of CRC samples. Scale bar: 100 μm. **K** Relationship between CPNE7 expression and CRC patients’ survival in HPA database. For (**G**), two-tailed paired Student’s *t*-test was used. For (**I**), data are shown as mean ± SD and two-tailed unpaired Student’s *t*-test was used. *p < 0.05, **p < 0.01, ***p < 0.001. Data are representative of at least three independent experiments.

To search novel important regulators in CRC, we analysed three online transcriptomic datasets (GSE142279, GSE166254 and GSE196006). 1,354 common differentially expressed genes (DEGs) were found in CRC tissues compared to normal tissues, among which 647 genes were up-regulated and 707 ones were down-regulated (Fig. 1A). Then, we performed Gene Ontology (GO) and Kyoto Encyclopedia of Genes and Genomes (KEGG) enrichment analysis on the common 1,354 DEGs. They were mainly enriched in pathways of hormone metabolism, receptor ligand activity, and G protein-coupled receptors (Fig. 1B, C). Next, we analysed 647 up-regulated common DEGs and select top 30 genes according to the *p*-value and fold change values (Fig. 1D). Notably, only the function of CPNE7, TTC26 and PACC1 in CRC has not been reported. We then focused on CPNE7 because its fold change value ranked top (Fig. 1D). We then confirmed its upregulation in colon adenocarcinoma (COAD) and rectum adenocarcinoma (READ) tissues according to UALCAN (Fig. 1E) and GEPIA database (Fig. 1F). We also collected 19 pairs of CRC samples. Consistently, CPNE7 was upregulated in most CRC samples compared to paired normal tissues (Fig. 1G). Importantly, the adjacent normal tissue, tumor tissue and liver metastatic tissue from the same patient were collected and used for RNA-sequencing. We observed that CPNE7 was highly expressed in tumor tissues compared to normal tissues and its level was further upregulated in liver metastatic tissues (Fig. 1H), which was confirmed by qRT-PCR and IHC analysis (Fig. 1I, J). Furthermore, CPNE7 high expression was associated with poor prognosis according to HPA database (Fig. 1K). In summary, CPNE7 is highly expressed in CRC and may be a prognostic biomarker.

### CPNE7 knockdown inhibits proliferation and migration of CRC cells

**Fig. 2:**
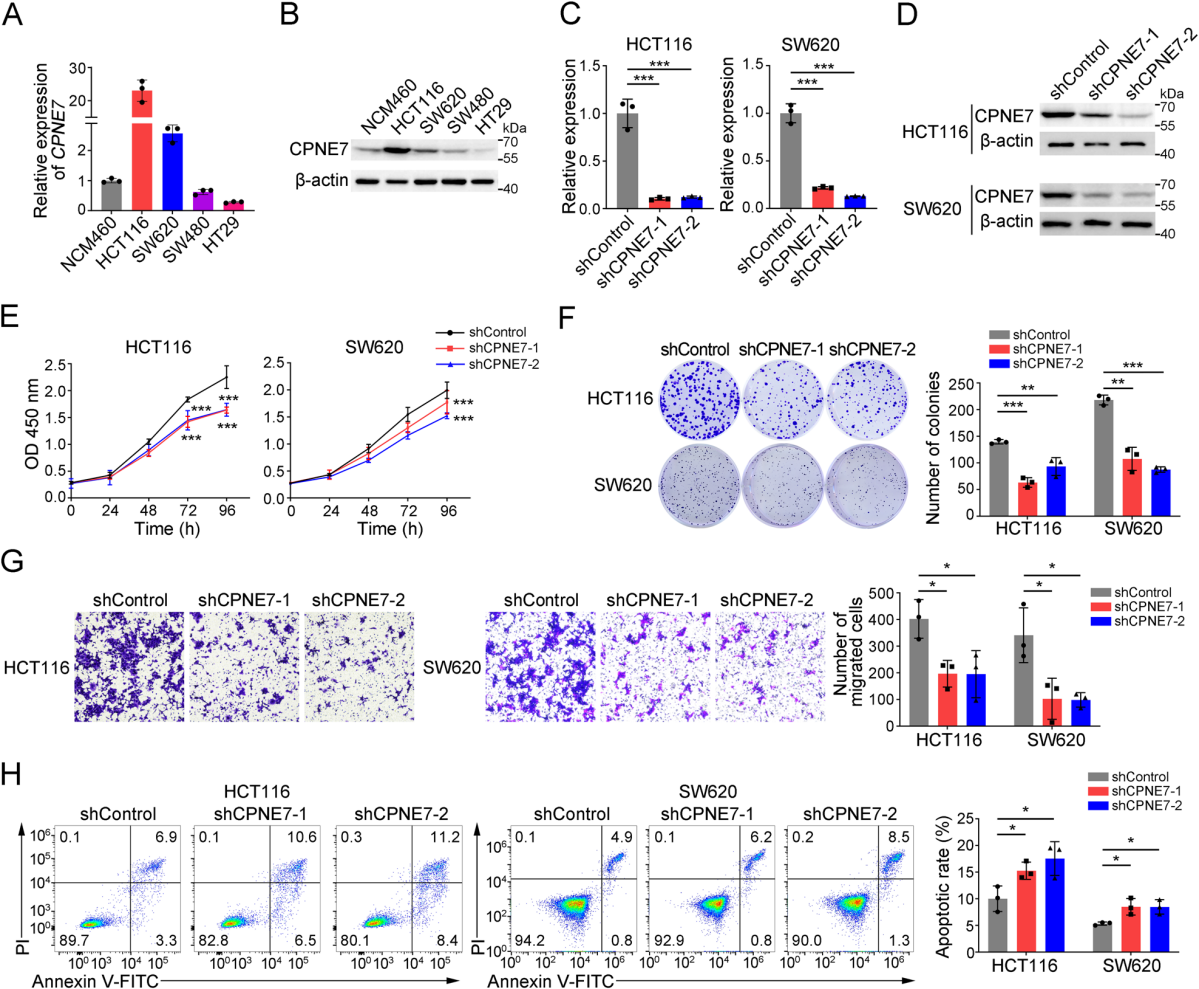
CPNE7 knockdown inhibits proliferation and migration of CRC cells. **A**, **B** Expression of mRNA (**A**) and protein (**B**) of CPNE7 in human normal colonic epithelial cell line NCM460 and human CRC tumor cell lines HCT116, SW620, SW480 and HT29. **C**, **D** Expression of CPNE7 at the mRNA level (**C**) and protein level (**D**) in HCT116 and SW620 cell lines. **E** Cell viability was measured by CCK-8 assay. **F** Colony formation assays of shControl and shCPNE7 CRC cells. Representative images are shown on the left, and the statistical analysis are is on the right. **G** Transwell assays of shControl and shCPNE7 CRC cells. Representative images are shown on the left, and the statistical analysis is shown on the right. **H** Apoptosis detection assays of shControl and shCPNE7 CRC cells. Representative images are shown on the left, and the statistical analysis of apoptotic rates (including early apoptosis and late apoptosis) is shown on the right. For (**A**–**H**), data are shown as mean ± SD and two-tailed unpaired Student’s *t*-test was used. *p < 0.05, **p < 0.01, ***p < 0.001. Data are re*p*resentative of at least three independent experiments.

To study the function of CPNE7 in CRC, we firstly examined its expression patterns in CRC cell lines (HCT116, SW620, SW480 and HT29) and human normal colonic epithelial cell line NCM460. We found CPNE7 was highly expressed in HCT116 and SW620 cells (Fig. 2A, B). Therefore, we decided to select HCT116 and SW620 cells for functional experiments. We first constructed CPNE7 stable knockdown cell lines using two independent shRNAs. CPNE7 level was significantly decreased in shCPNE7 cells compared to shControl cells (Fig. 2C, D). CCK-8 assay and colony formation assay showed that CPNE7 knockdown significantly impeded the growth of HCT116 and SW620 cells (Fig. 2E, F). Transwell assay and wound-healing assay indicated that CPNE7 knockdown significantly reduced the migration potential of tumor cells (Fig. 1G and Supplementary Fig. 1A, B). Additionally, we found that CPNE7 knockdown also increased the apoptotic ratio of tumor cells (Fig. 2H). We also generated a CPNE7 knockout HCT116 cell line using CRISPR/Cas9 technology (Supplementary Fig. 2A). We found that CPNE7 knockout suppressed the proliferation and migration of HCT116 cells (Supplementary Fig. 2B–E). Moreover, CPNE7 knockout promoted apoptosis in HCT116 cell line (Supplementary Fig. 2F). Collectively, CPNE7 knockdown inhibits proliferation and migration of CRC cells.

### CPNE7 knockdown inhibits the growth and metastasis of CRC cells in vivo

**Fig. 3:**
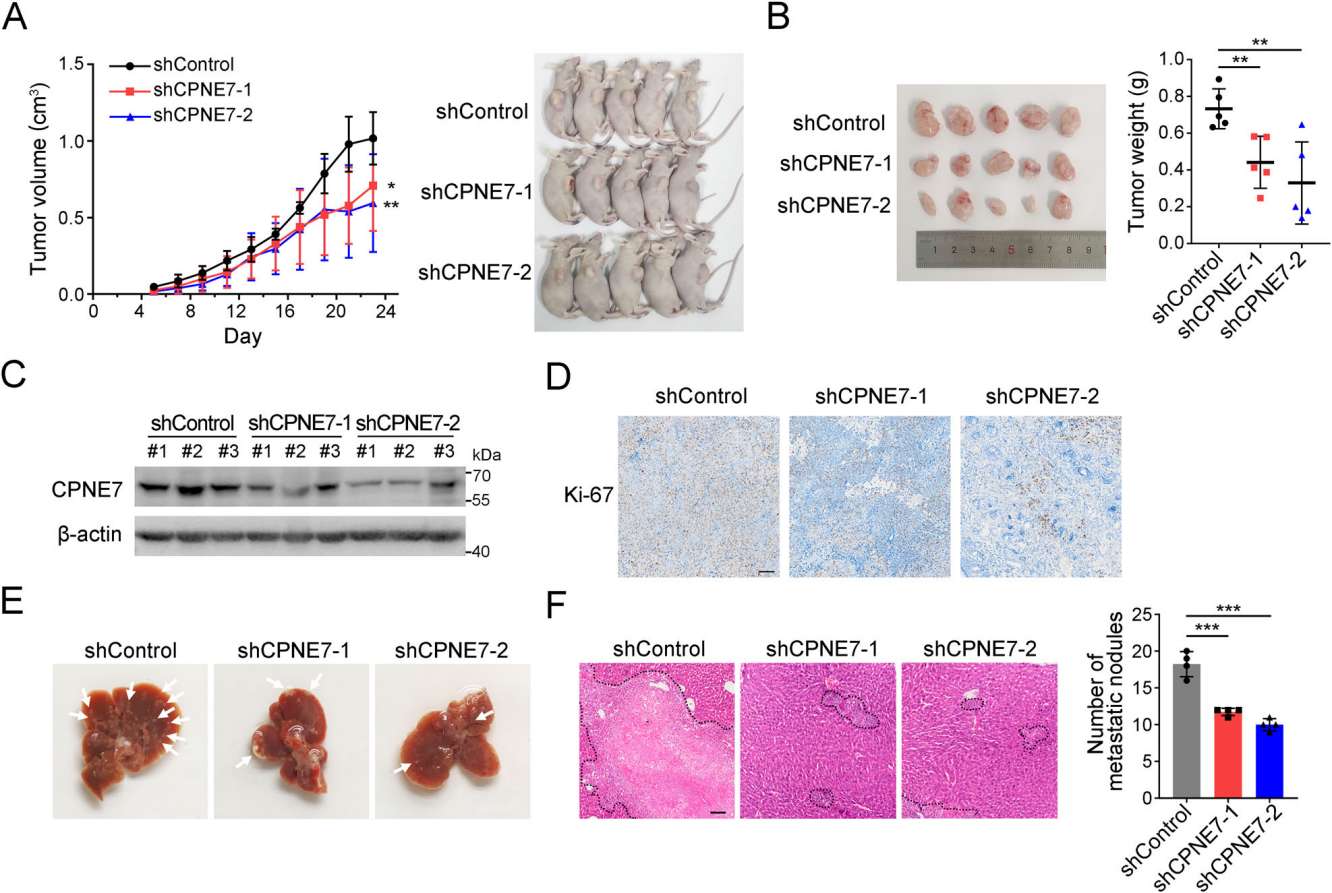
CPNE7 knockdown inhibits the growth and metastasis of CRC cells in vivo. **A** shControl and shCPNE7 HCT116 cells were injected into BALB/c-nude mice (*n* = 5 mice per group). Volume changes of xenograft tumors in nude mice are shown on the left, and mice images are shown on the right. **B** Tumor images are shown on the left, and statistical analysis for tumor weights is shown on the right. **C** Expression of CPNE7 in xenograft tumors were detected by Western blot. **D** IHC staining of Ki-67 in xenograft tumors in (**B**). Scale bar: 100 μm. **E** Representative images of the visible metastatic nodules in livers. (*n* = 4 mice per group). **F** Representative images of liver (**E**) histology stained with hematoxylin and eosin are shown on the left, and the statistical analysis is shown on the right. For (**A**– **F**), data are shown as mean ± SD and two-tailed unpaired Student’s *t*-test was used. *p < 0.05, **p < 0.01, ***p < 0.001. Data are re*p*resentative of at least three independent experiments.

To further explore the role of CPNE7 in vivo, cell derived xenograft (CDX) model was established by subcutaneous injecting CPNE7 knockdown HCT116 cells into BALB/c nude mice. We found that CPNE7 knockdown significantly suppressed tumors growth (Fig. 3A, B). Then, Western blot analysis confirmed reduced CPNE7 expression in the CPNE7 knockdown groups (Fig. 3C), which corresponded to the observed inhibition of tumor growth in these xenografts. IHC showed a remarkable decreased expression of Ki-67 (Fig. 3D), suggesting that the proliferation of CPNE7 knockdown CRC cells was inhibited. Additionally, we established metastasis model by tail vein injection. We observed a reduced number of metastatic nodules in mice injected with CPNE7 knockdown HCT116 cells compared to control mice (Fig. 3E, F). In summary, CPNE7 knockdown inhibits the growth and metastasis of CRC cells in vivo.

### CPNE7 overexpression promotes the proliferation and migration of CRC cells

We found that CPNE7 expression was the lowest in HT29 cells among four CRC cell lines (Fig. 2A). Thus, we selected HT29 cells to establish a stable CPNE7 overexpression cell line. We confirmed CPNE7 was overexpressed by Western blot (Fig. 3A). CCK-8 and colony formation assays indicated that CPNE7 overexpression enhanced the proliferation capacity of HT29 cells (Fig. 3B, C). Besides, transwell and wound-healing assays showed that CPNE7 overexpression resulted in increased migration of tumor cells (Fig. 3D, E). In summary, CPNE7 overexpression promotes the proliferation and migration of CRC cells.

**Fig. 4:**
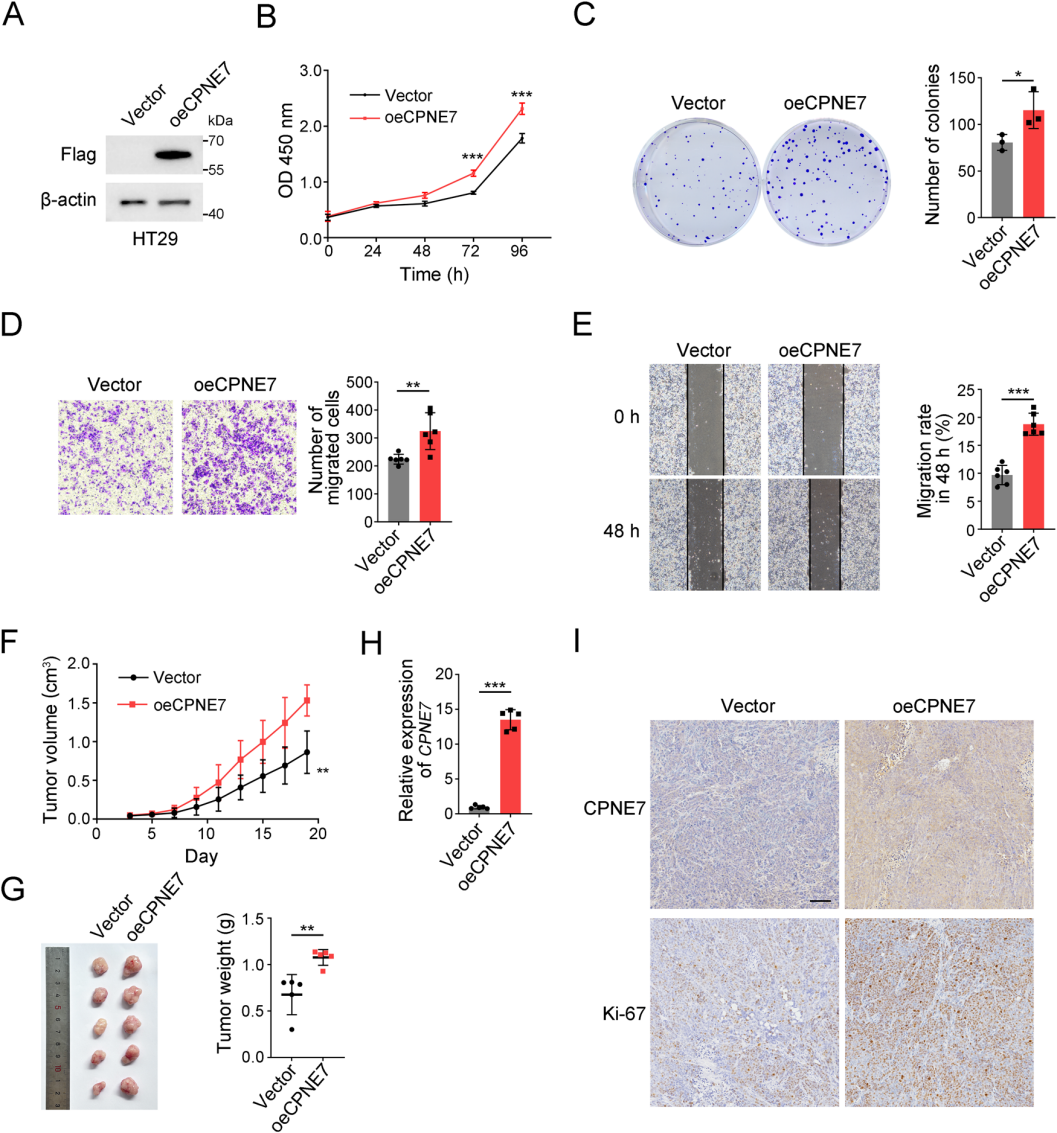
CPNE7 overexpression promotes the proliferation and migration of CRC cells. **A** CPNE7 overexpression HT29 cell line was established and verified by Western blot. **B** Cell viability was measured by CCK-8 assay. **C** Colony formation assays of Vector and oeCPNE7 HCT116 cells. Representative images are shown on the left, and the statistical analysis is shown on the right. **D** Transwell assays of Vector and oeCPNE7 HCT116 cells. Representative images are shown on the left, and the statistical analysis is shown on the right. **E** Wound-healing assays of Vector and oeCPNE7 HCT116 cells. Representative images are shown on the left, and the statistical analysis is shown on the right. **F** Vector and oeCPNE7 HT29 cells were injected into BALB/c-nude mice (*n* = 5 mice per group). Volume changes of xenograft tumors in nude mice are shown. **G** Tumor images are shown on the left, and statistical analysis for tumor weights is shown on the right. **H** Expression of CPNE7 in xenograft tumors were detected by qRT-PCR. **I** IHC staining of CPNE7 and Ki-67 in xenograft tumors in (**G**). Scale bar: 100 μm. For (**B**–**G**), data are shown as mean ± SD and two-tailed unpaired Student’s *t*-test was used. *p < 0.05, **p < 0.01, ***p < 0.001. Data are re*p*resentative of at least three independent experiments.

To further explore the tumorigenic capacity of CPNE7, we subcutaneously injected CPNE7 overexpressing HT29 cells into nude mice. The result showed that both the volume and growth rate of tumors in overexpression group were higher than those in the control group (Fig. 4F, G). Subsequent qRT-PCR (Fig. 4H) and IHC (Fig. 4I) assays confirmed the increased expression level of CPNE7 in the experimental group. Moreover, the elevated expression of Ki-67 in the overexpression group suggested a faster growth rate of tumors in this group (Fig. 4I). Taken together, these results illuminate that CPNE7 overexpression promotes tumor cells growth in vivo.

### CPNE7 interacts with NONO to promote ZFP42 transcription

**Fig. 5:**
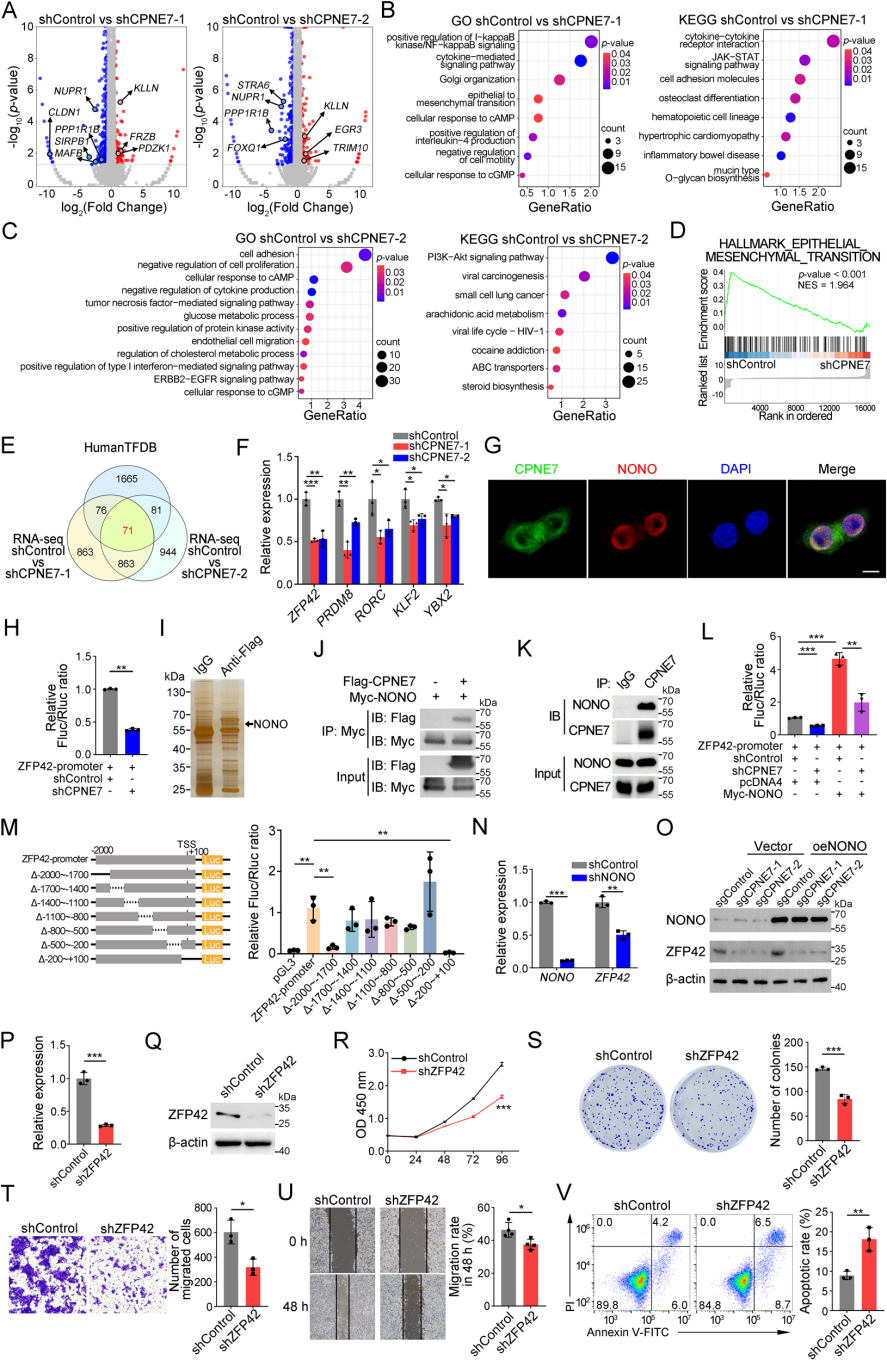
CPNE7 interacts with NONO to promote ZFP42 transcription. **A** Volcano plots of DEGs in shControl group vs shCPNE7 groups. **B**, **C** GO and KEGG analysis of DEGs from left volcano plot (**B**) and right volcano plot (**C**) in (**A**). **D** GSEA analysis of RNA-sequencing data from shControl group and shCPNE7-1 group. **E** Venn plot of HumanTFDB and DEGs in RNA-sequencing (|FoldChange | > 1.5). **F** 5 genes exhibiting the same trend in RNA-sequencing and qRT-PCR. **G** Immunofluorescence staining of CPNE7 and NONO in HCT116 cells. Scale bar: 10 μm. **H** Dual-luciferase reporter assay were performed in shControl and shCPNE7 293 T cells. **I** Proteins from the IP assay on SW480 cells were separated by SDS-PAGE and detected by silver staining. **J**, **K** Interaction between NONO and CPNE7 were verified in 293 T (**J**) and HCT116 (**K**) cells by Co-IP assay. **L** The effect of CPNE7 and NONO on ZFP42 promoter activity was detected by dual-luciferase reporter assay. **M** Left panel: schematic diagram of the structure of truncated ZFP42 promoter reporter plasmids. Right panel: Changes in luciferase activity following truncation at different positions of the ZFP42 promoter. **N** *ZFP42* mRNA expression were detected in shControl and shNONO SW620 cells. **O** Expression of NONO and ZFP42 were detected in CPNE7 knockout HCT116 cells by Western blot. **P**, **Q** mRNA expression level (**P**) and protein expression level (**Q**) of ZFP42 in shControl and shZFP42 HCT116 cells. **R** Cell viability was measured by CCK-8 assay. **S** Colony formation assays of shControl and shZFP42 HCT116 cells. Representative images are shown on the left, and the statistical analysis is shown on the right. **T** Transwell assays of shControl and shZFP42 HCT116 cells. Representative images are shown on the left, and the statistical analysis is shown on the right. **U** Wound-healing assays of shControl and shZFP42 HCT116 cells. Representative images are shown on the left, and the statistical analysis for migration rates is shown on the right. **V** Apoptosis detection assays of shControl and shZFP42 HCT116 cells. Representative images are shown on the left, and the statistical analysis for apoptotic rates (including early apoptosis and late apoptosis) is shown on the right. For (**F**–**V**), data are shown as mean ± SD and two-tailed unpaired Student’s *t*-test was used. *p < 0.05, **p < 0.01, ***p < 0.001. Data are representative of at least three independent experiments.

To investigate the molecular mechanism of CPNE7 in regulating CRC progression, we performed RNA-sequencing using shControl and shCPNE7 HCT116 cells. Of note, many classical oncogenes were downregulated after CPNE7 knockdown, such as CLDN1, FOXQ1 and MAFB (Fig. 5A). Next, the DEGs were utilized for GO and KEGG analysis. We noticed that several cancer-related pathways were enriched, including JAK-STAT pathway, ERBB2 − EGFR and PI3K-AKT signaling (Fig. 5B, C). Also, gene-set enrichment analysis (GSEA) indicated EMT was negatively associated with shCPNE7 group (Fig. 5D), supporting its critical role on tumor metastasis. Transcription factors (TFs) play a vital role in tumorigenesis and progression in many ways [27–29]. Interestingly, after CPNE7 silencing, a total of 71 TFs were aberrantly expressed according to RNA-sequencing result (Fig. 5E). Importantly, only 54 TFs showed consistent change trends (Supplementary Fig. 3 A). To validate the RNA-sequencing results, we performed qRT-PCR using two shCPNE7 HCT116 cell lines and found that there were only 5 TFs (ZFP42, PRDM8, RORC, KLF2 and YBX2) whose change was consistent with RNA-sequencing results (Fig. 5F and supplementary Fig. 3B). Of above 5 TFs, ZFP42 downregulation was the most obvious (Fig. 5F). We observed CPNE7 was also distributed in the nucleus of cancer cells (Fig. 5G), implying it may regulate ZFP42 transcription. Due to the decrease in ZFP42 expression following CPNE7 knockdown, we speculated that CPNE7 might regulate the transcription of ZFP42. To validate it, we performed luciferase reporter assay. Consistently, CPNE7 knockdown suppressed the luciferase activity of the ZFP42-promoter reporter (Fig. 5H). To further explore how CPNE7 regulated ZFP42 transcription, we performed mass spectrometry analysis. We identified that CPNE7 was associated with NONO (Fig. 5I and Supplementary Fig. 4), which was verified by co-immunoprecipitation (Co-IP) assays (Fig. 5J, K). We observed CPNE7 and NONO were colocalized in the nucleus of cancer cells (Fig. 5G and Supplementary Fig. 5), implying they may jointly regulate the transcription of ZFP42. Expectedly, the interaction between NONO and CPNE7 could enhance the transcription efficiency of ZFP42 (Fig. 5L). To further determine the binding sites of CPNE7 with the ZFP42 promoter, we constructed the truncated promoter into reporter plasmids. We found that CPNE7 might enhance ZFP42 transcription by binding to sites between -2000 to -1700 bp and -200 to +100 bp at the ZFP42 transcription start site (TSS) (Fig. 5M). NONO belongs to the family of Drosophila Behavioral/Human Splicing (DBHS) proteins that can bind to DNA, RNA and proteins [30]. NONO can directly affect gene transcription [31]. NONO silencing also reduced ZFP42 expression (Fig. 5N). However, the role of NONO in promoting ZFP42 expression is dependent on CPNE7 (Fig. 5O). To explore the role of ZFP42 in CRC, we established ZFP42 stable knockdown HCT116 cell line (Fig. 5P, Q). CCK-8 and colony formation assays showed ZFP42 knockdown dramatically reduced proliferation of tumor cells (Fig. 5R, S). Besides, transwell assay and wound-healing assay showed ZFP42 knockdown inhibited the migration of tumor cells (Fig. 5T, U). In addition, ZFP42 knockdown promoted apoptosis of CRC cells (Fig. 5V). In summary, these data provide evidences that ZFP42 knockdown inhibits proliferation and migration of CRC cells.

### Targeting CPNE7 or blocking its interaction with NONO impedes CRC growth in vivo

**Fig. 6:**
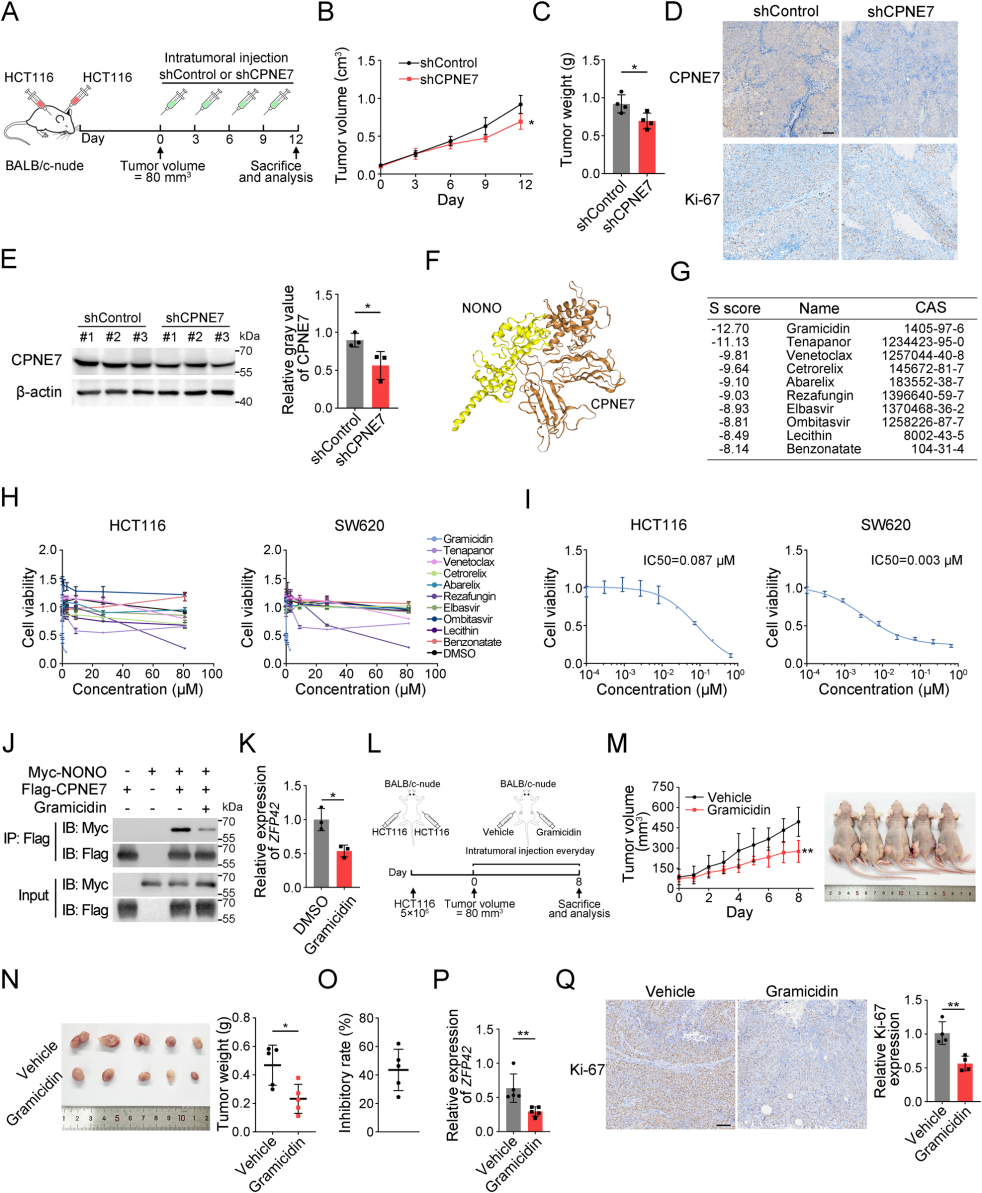
Targeting CPNE7 or blocking its interaction with NONO impedes CRC growth in vivo. **A** Schematic of CPNE7 shRNA intratumoral injection. **B**, **C** Tumor volumes (**B**) and tumor weights (**C**) of two groups. (*n* = 4 tumors per group). **D** IHC staining of CPNE7 and Ki-67 in tumors in (**A**). Scale bar: 100 μm. **E** Protein expression level of CPNE7 in (**A**) was detected by Western blot. **F** Schematic of protein docking between CPNE7 and NONO. **G** Top 10 compounds with the lowest S score obtained from virtual screening. **H** Compound toxicity was tested by CCK-8 assay. **I** IC50 determination of Gramicidin in HCT116 and SW620 cells. **J** Disruption of Gramicidin on CPNE7-NONO interaction was verified by Co-IP assay. The concentration of Gramicidin was 8 nM. **K** The expression of *ZFP42* mRNA in SW620 was detected after treatment with Gramicidin. **L** Schematic of Gramicidin intratumoral injection. **M** Volume changes of subcutaneous tumors in nude mice are shown on the left, and mice image are shown on the right (*n* = 5 tumors per group). **N** Tumor images are shown on the left, and statistical analysis for tumor weights is shown on the right. **O** Tumor inhibition rate. **P** The expression of *ZFP42* mRNA in tumors was detected by qRT-PCR. **Q** IHC staining of Ki-67 in tumors in (**M**). Scale bar: 100 μm. For (**B**–**Q**), data are shown as mean ± SD and two-tailed unpaired Student’s *t*-test was used. *p < 0.05, **p < 0.01, ***p < 0.001. For (**H**–**Q**), data are representative of at least three independent experiments.

To evaluate the therapeutic potential of CPNE7 in CRC, we injected CPNE7 shRNA plasmid intratumorally into tumors of nude mice (Fig. 6A). Tumor volume and weight were significantly reduced after shCPNE7 injection (Fig. 6B, C). We also found that proliferative tumor cells were reduced by CPNE7 knockdown (Fig. 6D, E), supporting its therapeutic ability.

We then performed virtual screening to search small molecule compounds which interfere with the interaction between CPNE7 and NONO. Of the 2,992 compounds, only 10 candidates were selected based on their highest S score (Fig. 6F, G). We then evaluated their antitumor potential using the CCK-8 assay and identified gramicidin based on its lowest IC50 value (Fig. 6H, I). Furthermore, the Co-IP assay showed that gramicidin can actually inhibit the interaction between CPNE7 and NONO (Fig. 6J). In addition, gramicidin can also inhibit the expression of ZFP42 (Fig. 6K). To further verify the role of gramicidin in vivo, we perform intratumoral injection assay (Fig. 6L). As expected, gramicidin significantly inhibited tumor growth (Fig. 6M–O) and the expression of ZFP42 (Fig. 6P). Consistently, Ki-67 expression was remarkable decreased after gramicidin intratumoral injection (Fig. 6Q). In conclusion, the CPNE7-NONO axis may serve as a new potential therapeutic target.

## Discussion

CRC is a major public health problem worldwide due to high morbidity and mortality. However, the underlying molecular mechanism remains unclear. It is important to discover new therapeutic targets and diagnostic markers for CRC treatment. In this study, upregulation of CPNE7 was observed in CRC tissues, which could be a prognostic biomarker. Loss-of-function or gain-of-function assays demonstrated that CPNE7 is crucial for CRC growth and metastasis. In mechanism, CPNE7 associates with NONO to initiate ZFP42 transcription, leading to tumorigenesis. Furthermore, we demonstrated that targeting CPNE7-NONO axis may be a novel strategy for CRC interventions.

Previous studies on the function of CPNE7 are mainly focused on how it regulates tooth development [10, 11, 14–16]. Its role in tumorigenesis is gradually being discovered. Kong H et al. discovered that CPNE7 can promote the metastasis of CRC by regulating EMT [32]. Xu W et al. identified that CPNE7 can modulate the chemoresistance of CRC through upregulation of ATG9B [33]. Yu T. et al. found that CPNE7 is distributed in the cytoplasm and interacted with PKM2 to enhance the phosphorylation of AKT and ERK, leading to activation of MAPK signaling [34]. Overall, the above studies have shown that CPNE7 plays a pivotal role in the progression of CRC. We also found CPNE7 promoted CRC proliferation and migration in vitro. Furthermore, we further demonstrated that CPNE7 initiated CRC metastasis in vivo and further revealed its therapeutic potential. Mechanistically, we found that CPNE7 was largely expressed in the nucleus of CRC cells and bound to NONO to directly regulate gene transcription, suggesting that CPNE7 was an important multifunctional protein. Thus, more exploration will be meaningful to define how CPNE7 works in tumorigenesis.

Emerging studies have reported that NONO is involved in the pathogenesis of several malignant tumors [35–37]. Besides, NONO is extensively involved in the regulation of gene expression by interacting with DNA, RNA and proteins [31, 38]. It has been reported that NONO can interact with STAT3 proteins to increase their stability and promote transcription of STAT3 target genes in breast cancer cells [39]. Although NONO has been demonstrated to be an oncogene in CRC [40], how it initiates tumor progression remains unclear. Here we found that NONO interacted with CPNE7 by mass spectrum. We also pointed out that NONO cooperated with CPNE7 to promote ZFP42 transcription and cancer growth and metastasis, which clearly defined its functional mechanism. Certainly, how NONO-CPNE7 organizes the transcription complex to initiate transcription remains to be investigated in the future.

It has been reported that ZFP42 is widely considered as a stem cell marker [22, 23] and its expression is associated with increased pluripotency in many pluripotent self-renewing cells [24]. However, the role of ZFP42 in tumorigenesis is controversial. ZFP42 acts as an oncogene in prostate cancer, cervical cancer and glioblastoma multiforme [24, 25, 41], whereas ZFP42 is identified as a tumor suppressor gene in hepatocellular carcinoma and renal cell carcinoma [26, 42]. Therefore, ZFP42 may be a multifunctional transcription factor that can promote either or inhibit carcinogenesis. To date. there is no evidence to implicate ZFP42 in the development and progression of CRC, and our research suggested that ZFP42 was a key gene that may promote progression of CRC. Although we found that transcription of ZFP42 could be promoted by CPNE7, the downstream pathways and specific molecular targets of ZFP42 remain to be elucidated.

Computer-aided drug design is widely used in drug research and development [43]. A number of studies have reported that virtual screening can be used for the development of inhibitors and agonists [44, 45]. Here, we identified gramicidin as an inhibitor of the CPNE7-NONO interaction by virtual screening. Previous studies have shown gramicidin has an antitumor effect in ovarian cancer, cholangiocarcinoma and gastric cancer [46–48]. Whether and how gramicidin affects CRC is unclear. Our study demonstrated that gramicidin can inhibit CRC cell growth in vitro and in vivo by interfering with the interaction between CPNE7 and NONO, supporting its ability as a novel therapeutic target.

## Methods

### Antibodies and reagents

The following antibodies were used in our study: Anti-CPNE7 antibody (Cat# bs-14030R, Bioss), Anti-CPNE7 Polyclonal antibody (Cat# 17396-1-AP, Proteintech), Anti-p54/nrb (NONO) antibody (Cat# sc-376865, Santa Cruz), Anti-Rex-1 (ZFP42) antibody (Cat# sc-376865, Santa Cruz), Anti-β-actin antibody (Cat# A5441, Sigma), Anti-Flag M2 antibody (Cat# F1804, Sigma), Anti-Myc Antibody (Cat# sc-40, Santa Cruz), Anti-mouse HRP (Cat# ZB-2305, Zsbio), Anti-rabbit HRP (Cat# ZB-2301, Zsbio), Goat anti-Mouse IgG (H + L) Alexa Fluor™ 594 (Cat# R37121, Invitrogen), Goat anti-Rabbit IgG (H + L) Alexa Fluor™ 647 (Cat# A-21245, Invitrogen). Gramicidin (CAS# 1405-97-6), Tenapanor(CAS# 1234423-95-0), Venetoclax (CAS# 1257044-40-8), Cetrorelix (CAS# 145672-81-7), Abarelix (CAS# 183552-38-7), Rezafungin, (CAS# 1396640-59-7), Elbasvir (CAS# 1370468-36-2), Ombitasvir (CAS# 1258226-87-7), Lecithin (CAS# 8002-43-5) and Benzonatate (CAS# 104-31-4) were purchased from TargetMol.

### Quantitative real-time PCR (qRT-PCR) and RNA-sequencing

RNA was extracted from tissues or cells by RNA isolater Total RNA Extraction Reagent (Cat# R401-01, Vazyme). For qRT-PCR, 1 μg of total RNA was reverse transcribed into complementary DNA (cDNA) by HiScript II Q RT SuperMix for qPCR (Cat# R223-01, Vazyme). Then cDNA was used as templates for quantitative analysis of mRNA. The primer sequences are listed in Supplementary Table 1. For RNA-sequencing, RNA libraries preparation, quality examination and RNA-sequencing were performed by BGI (Shenzhen, China). For visualization of RNA-sequencing reads in IGV genome browser, the bamCoverage function of deepTools with normalization to RPGC was used to generate the bigwig files. GO and KEGG analysis were performed by ClusterProfiler (v4.6.2). Gene set enrichment analysis (GSEA) was performed by MSigDB collections (https://www.gsea-msigdb.org/gsea/msigdb/index.jsp) through the GSEA in ClusterProfiler. Normalized enrichment scores and empirical *p*-values were estimated using default parameters, and multiple testing correction was carried out using the Benjamini–Hochberg method.

### Immunohistochemistry (IHC)

All tissues were fixed with 4% paraformaldehyde and embedded in paraffin. After dewaxing and rehydration, slides were heated in boiling antigen retrieval buffer. After returning to room temperature (RT), tissues were incubated with 3% H_2_O_2_ for 10 min at RT. Subsequently, tissues were blocked with 10% donkey serum, followed by incubating with primary antibody overnight at 4°C. The next day, tissues were incubated with secondary antibody at RT for 30 min. After that, tissues were stained with DAB followed by hematoxylin staining for nuclei. After dehydrated with ethanol and xylene, the slide was covered with a cover slip.

### Human samples and cell culture

CRC samples were collected from The First Affiliated Hospital of Zhengzhou University with informed consent, according to the Institutional Review Board approval. All experiments were approved by Ethics Committee of Zhengzhou University. The human normal colonic epithelial cell line NCM460 was purchased from BLUEFBIO (Cat# BFN608006385). The human cell line 293 T and the human CRC tumor cell lines HCT116, SW620, SW480, HT29 were provided by Pingping Zhu (School of Life Sciences, Zhengzhou University). All cells were maintained in humidified incubator at 37°C with 5% CO_2_. All cells were grown in Dulbecco’s Modified Eagle Medium (Cat# PM150210, Procell) supplemented with 10% FBS (Cat# S711-001S, Lonsera), 100 μg/mL penicillin and 100 U/mL streptomycin (Cat# C0222, Beyotime). All cell lines were tested for mycoplasma contamination and authenticated using an STR profiling.

### Lentiviral infection

293 T cells were cultured to reach 60-80% confluence and transfected using jet PRIME (Cat# 101000046, Polyplus). Ratio of Vectors (pLVX-IRES-ZsGreen1, pSicoR puro or lentiCRISPRv2), psPAX2 (Cat# 12260, Addgene) and pMD2.G (Cat# 12259, Addgene) was 4:3:2. After 4 h incubation, cells were replaced with new medium and cultured another 36–48 h. After that, supernatants containing virus were collected followed by filtered with 0.45 μm filter and added to target cells with polybrene (Cat# 40804ES76, YEASEN) for infection.

### Plasmid construction

For construction of knockdown vectors, we connected shRNA to pSicoR-puro (Cat# 12084, Addgene). shRNA primers are listed in Supplementary Table 2. For construction of knockout vectors, we connected sgRNA to lentiCRISPRv2 (Cat# 98292, Addgene). sgRNA primers are listed in Supplementary Table 3. For construction of overexpression vectors, we cloned CPNE7 or NONO coding sequence into pLVX-IRES-ZsGreen1 (Cat# 632187, Takara) and pcDNA™4/myc-His B (Cat# V86320, Invitrogen) respectively. Overexpression primers are listed in Supplementary Table 4.

### Western blot

Cells were lysed with RIPA (Cat# P0013B, Beyotime) for proteins extraction, then the protein was quantified using BCA protein quantification kit (Cat# P0012, Beyotime). After boiling the samples for 10 min, SDS-PAGE electrophoresis was used to separate proteins with 80 V for concentrated gels and 120 V for separated gels. Subsequently, proteins were transferred to PVDF membranes (Cat# IPVH00010, Millipore) at 300 mA for 120 min. After incubated with 8% skimmed milk for 60 min at RT, PVDF membranes were incubated with primary antibodies at 4°C overnight. Next day, PVDF membranes were incubated with secondary antibody at RT for 1 h. At last, ECL detection kit (Cat# P0018S, Beyotime) was used to detect protein.

### CCK-8 assay

3000 cells were seeded into 96-well plate and then cultured in cell incubator. 10 μL of CCK-8 (Cat# K1018, APExBio) was added in cells at 0 h, 24 h, 48 h, 72 h and 96 h, then the 96-well plates were cultured in cell incubator for 1 h. The absorbance at 450 nm was measured.

### Colony formation assay

500 cells were added to 6-well plate and cultured in cell incubator about 10 days. After that, the cells were fixed with 4% PFA and stained with 1‰ crystal violet. At last, the 6-well plates were dried and scanned into pictures for analysis.

### Wound-healing assay

Cells were seeded into 6-well plates and achieved 100% confluence after overnight culture. Next day, the 10 μL pipette tip was used to draw a straight line on the cells, then photographed the line as 0 h. Subsequently, the culture medium was replaced by serum-free medium, and the cells were continued to be cultured for 48 h before photographed again. The migration rate of the cells was calculated as (Width 0 h– Width 48 h) / Width 0 h.

### Transwell assay

Cells were diluted to 1 × 10^5^ cells/mL in serum-free DMEM. Subsequently, 200 μL cell suspension was added to the upper chamber, and 600 μL medium containing 20% FBS was added to the lower chamber (Cat# 3422, Corning). After incubation for 24 h, the transwell chambers were fixed with 4% PFA and stained with 1‰ crystal violet.

### Apoptosis assay

Apoptosis assay was performed using Annexin/PI double staining kit (Cat# 40302ES50, Yeasen) according to the manufacturer’s instructions. Briefly, cells were collected and washed with PBS. After discarding the supernatant, cells were stained with 10 μg/mL of Annexin V-FITC and 5 μg/mL of PI. Then cells were measured with a flow cytometer (BD Accuri™ C6 Plus, BD Bioscience).

### Animal experiments

The animal experiments were approved by Ethics Committee of Zhengzhou University. Five to six weeks old BALB/c-nude female mice were purchased form SPF (Beijing) Biotechnology Co., Ltd. No statistical methods were applied to choose the number of mice. For this analysis; blinding was not conducted. The animals were grouped by the same age and gender, and were randomly allocated to experimental groups. For CDX assay, HCT116 tumor cells (3 × 10^6^ cells per mouse) were injected subcutaneously into the right flank of mice for 23 days. For metastasis assay, HCT116 tumor cells (2 × 10^6^ cells per mouse) were injected into the tail vein of mice for 35 days. For intratumoral injection experiments, HCT116 tumor cells (3 × 10^6^ cells per mouse) were injected subcutaneously into both flanks of mice for 15 days. The tumor volume was monitored every 1–3 days using the formula *V* = (length) × (width)^2^ × π/6. Mice were euthanized at the end of the experiment.

### Immunofluorescence staining

Cells were inoculated on slides overnight, fixed with 4% paraformaldehyde at RT for 1 h. After permeabilized in 1% Triton X-100 at RT for 1 h, cells were blocked with 10% donkey serum (Cat# S9100, Solarbio) at RT for 1 h. Next, cells were incubated with primary antibody overnight at 4°C. After washing 3 times with PBS, cells were incubated with secondary antibody at RT for 1 h. At last, the slides were sealed with antifading mounting medium (Cat# S2100, Solarbio).

### Immunoprecipitation (IP) and co-immunoprecipitation (Co-IP)

Cells were lysed with RIPA buffer (Cat# P0013B, Beyotime) containing PMSF (Cat# P0100, Solarbio) on ice for 30 min. After centrifugation at 12,000 g for 20 min at 4°C, 40 μL supernatant was collected as input, and the remaining supernatants were incubated with 2 μg antibody for IP overnight at 4°C. The next day, the supernatants were incubated with Protein A/G beads (Cat# IF0001, Engibody) for 2 h at 4°C followed by centrifugation at 3000 g for 5 min at 4°C. The beads were collected and washed with pre-cooled RIPA buffer followed by boiled with 40 μL 2 × SDS loading buffer for 10 min. For IP, the samples were subjected to SDS-PAGE gels and detected by silver staining. For Co-IP, the samples were subjected to SDS-PAGE gels and detected by Western blot.

### Silver staining and mass spectrometry

Silver staining was performed using Fast Silver Stain Kit (Cat# P0017S, Beyotime) according to the manufacturer’s instructions. Differential bands were collected for mass spectrometry analysis (Shanghai OE Biotech Co., Ltd).

### Dual-Luciferase reporter assay

Promoter of ZFP42 was cloned into pGL3-basic plasmid, and co-transfected 293 T cells with the internal reference plasmid pRL-TK, CPNE7 knockdown plasmid and NONO overexpression plasmid. The plasmids ratio of pGL3-basic versus pRL-TK was 100:1. The luciferase activity was detected by dual-Luciferase reporter gene assay kit (Cat# RG027, Beyotime) according to the manufacturer’s instructions. The ZFP42 promoter sequence and the sequences within it that may bind to CPNE7 are listed in Supplementary Table 5.

### Virtual screening

PDB format file of NONO protein was downloaded from The Protein Data Bank (https://www.rcsb.org/). Structure of CPNE7 was predicted by SWISS-MODEL (https://swissmodel.expasy.org/). HDOCKSERVER (http://hdock.phys.hust.edu.cn) was used for predicting the structure of protein-protein complex. Molecular Operating Environment (MOE) was used for docking proteins and compounds. Compounds were docked by triangle matcher placement with London dG scoring for 30 poses and induced fit refinement with GBVI/WSA dG scoring for 5 poses. Finally, compounds were selected based on S score, molecular weight, research background and other factors.

### Statistics analysis

The data from both groups were normally distributed, and *p*-values between the two groups were calculated using two-tailed paired or unpaired Student’s *t*-tests when the variances were similar. Data that did not conform to normal distribution were analyzed using nonparametric tests. Data with dissimilar variances between groups were analyzed using Welch’s correction. *p* < 0.05 was considered significant, *p < 0.05, **p < 0.01, ***p < 0.001, n.s., no significance. No statistical method was used to determine sample size. No data were excluded from the analyses. All values were expressed as mean values ± SD. For all panels, at least three independent experiments were performed with similar results, and representative experiments are shown. Data were analysed by GraphPad Prism 5. Images were composed by Adobe Photoshop CC 14.

### Accession numbers

Raw RNA-sequence data have been deposited in the NCBI Sequence Read Archive PRJNA1179681 and PRJNA1179711.

## Supplementary information

Supplementary information

Uncropped Western blots-1

Uncropped Western blots-2

## Data Availability

All data are available upon reasonable request.
