# Supplementary material for: CPNE7 promotes colorectal tumorigenesis by interacting with NONO to initiate ZFP42 transcription
Source: Cell Death Dis. 2024 Dec 18;15(12):896. doi: 10.1038/s41419-024-07288-z (PMC11655532; doi:10.1038/s41419-024-07288-z)
Supplement: Supplementary file 1 — Supplementary information [file 41419_2024_7288_MOESM1_ESM.docx]

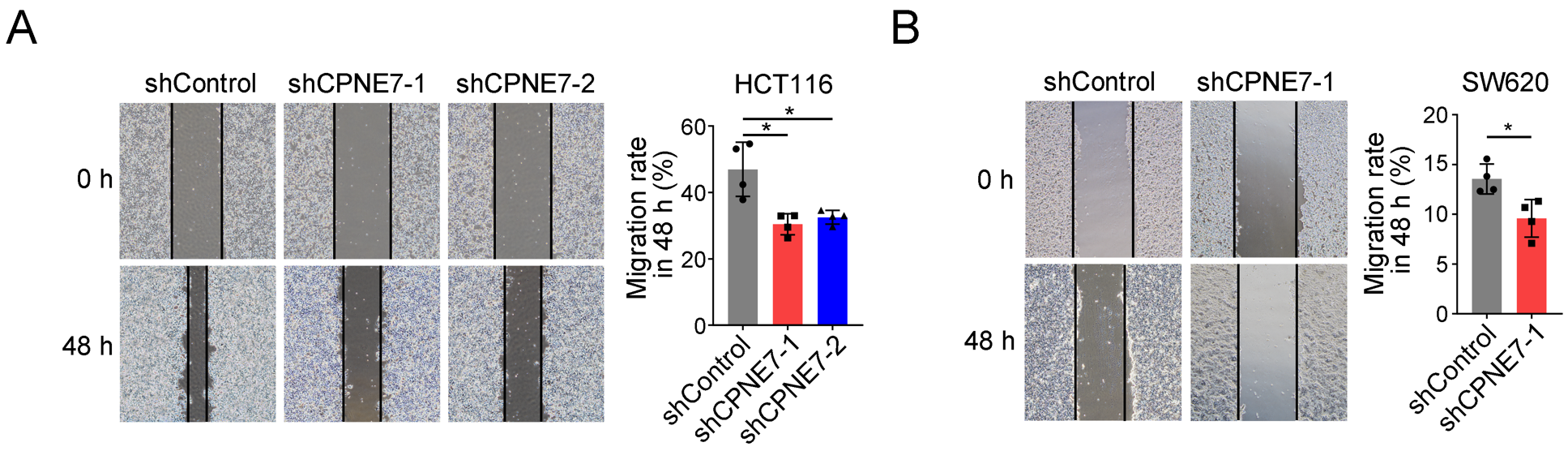


**Supplementary Figure 1. CPNE7 knockdown inhibits CRC migration.** (A, B) Wound-healing assays of HCT116 (A) and SW620 (B) cells. Representative images are shown on the left, and the statistical analysis for migration rates is shown on the right. For (A, B), data are shown as mean ± SD and two-tailed unpaired Student’s t-test was used. **p* < 0.05. Data are representative of at least three independent experiments.


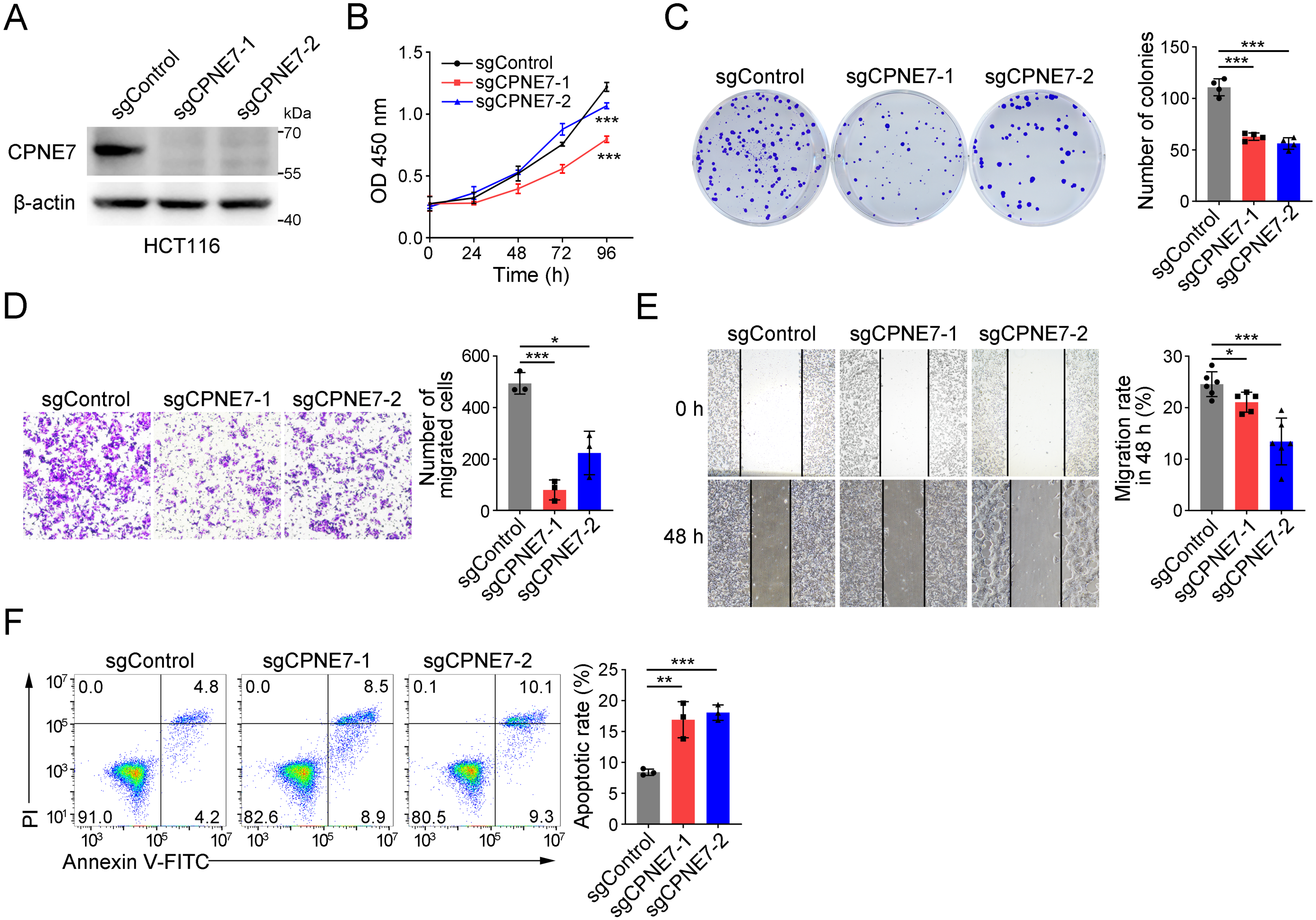


**Supplementary Figure 2. CPNE7 knockout inhibits CRC proliferation and migration.** (A) CPNE7 protein levels in sgControl and sgCPNE7 HCT116 cells were examined by Western blot. (B) Cell viability was measured by CCK-8 assay. (C) Colony formation assays of sgControl and sgCPNE7 HCT116 cells. Representative images are shown on the left, and the statistical analysis is shown on the right. (D) Transwell assays of sgControl and sgCPNE7 HCT116 cells. Representative images are shown on the left, and the statistical analysis is shown on the right. (E) Wound-healing assays of sgControl and sgCPNE7 HCT116 cells. Representative images are shown on the left, and the statistical analysis for migration rates is shown on the right. (F) Apoptosis detection assays of sgControl and sgCPNE7 HCT116 cells. Representative images are shown on the left, and the statistical analysis for apoptotic rates (including early apoptosis and late apoptosis) is shown on the right. For (B, C, D, E, F,), data are shown as mean ± SD and two-tailed unpaired Student’s t-test was used. **p* < 0.05, ***p* < 0.01, ****p* < 0.001. Data are representative of at least three independent experiments.


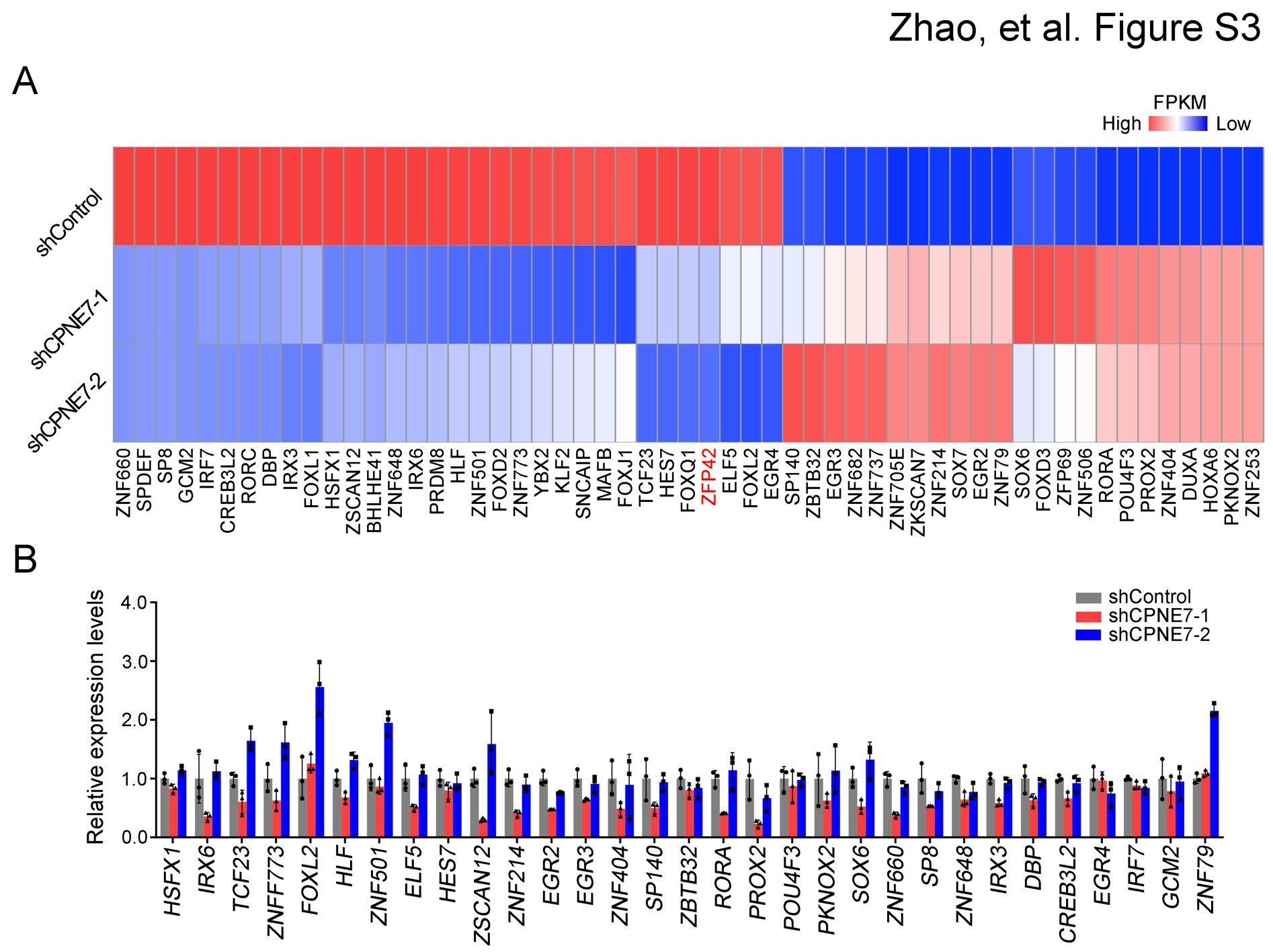
 **Supplementary** **Figure 3. RNA-sequencing after CPNE7 silencing.** (A) 54 genes showed consistent change trend in both shCPNE7 RNA-sequencing groups. FPKM: Fragments Per Kilobase Million. (B) 31 genes showed unconsistent change trend in RNA-sequencing and qRT-PCR assays. For (B), data are shown as mean ± SD. Data are representative of at least three independent experiments.


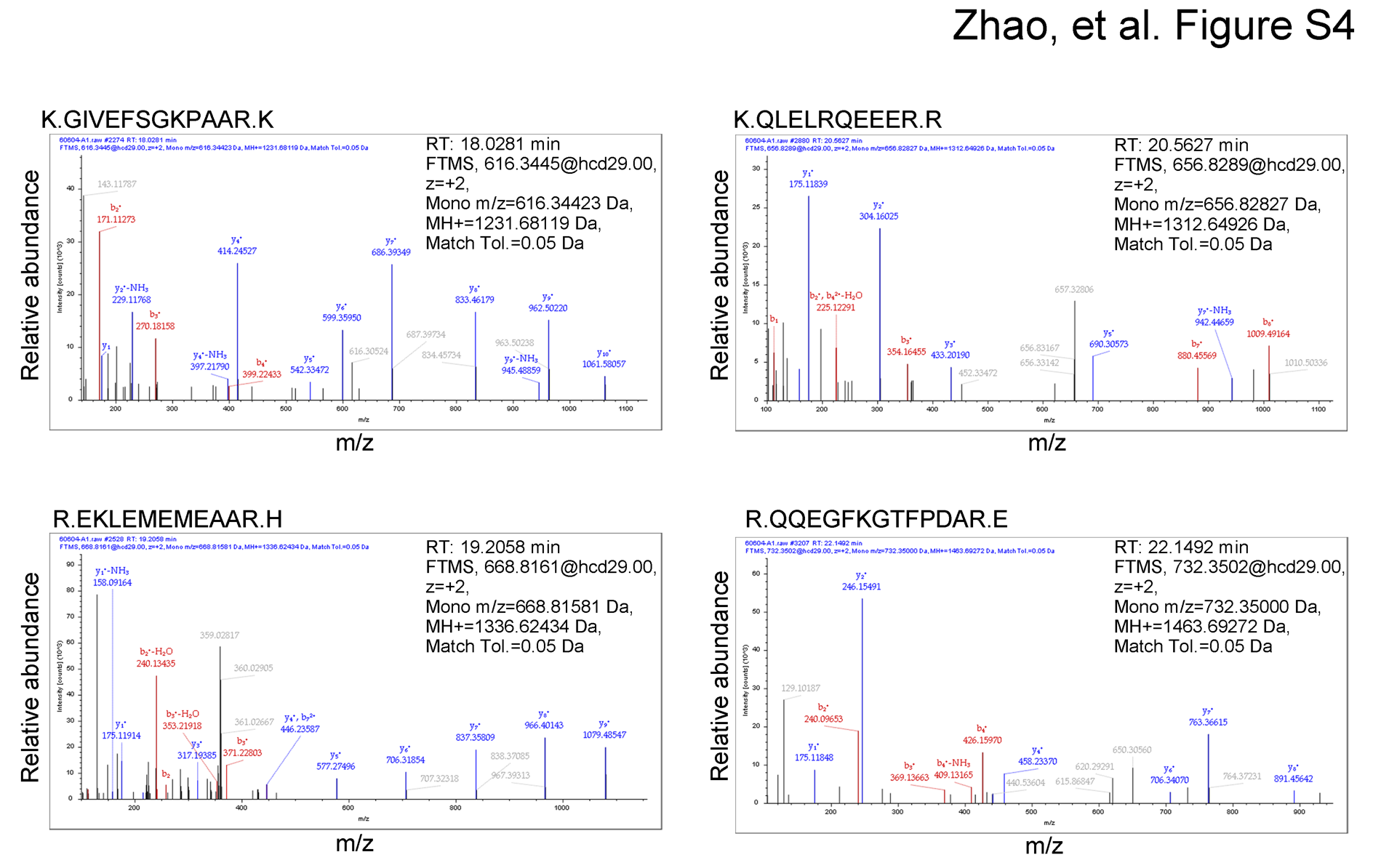


**Supplementary Figure 4. Mass spectrometry profiles for NONO.** Captured peptide sequences of mass spectrometry profiles for NONO, corresponding peptide sequences are listed on the top of corresponding graphs.


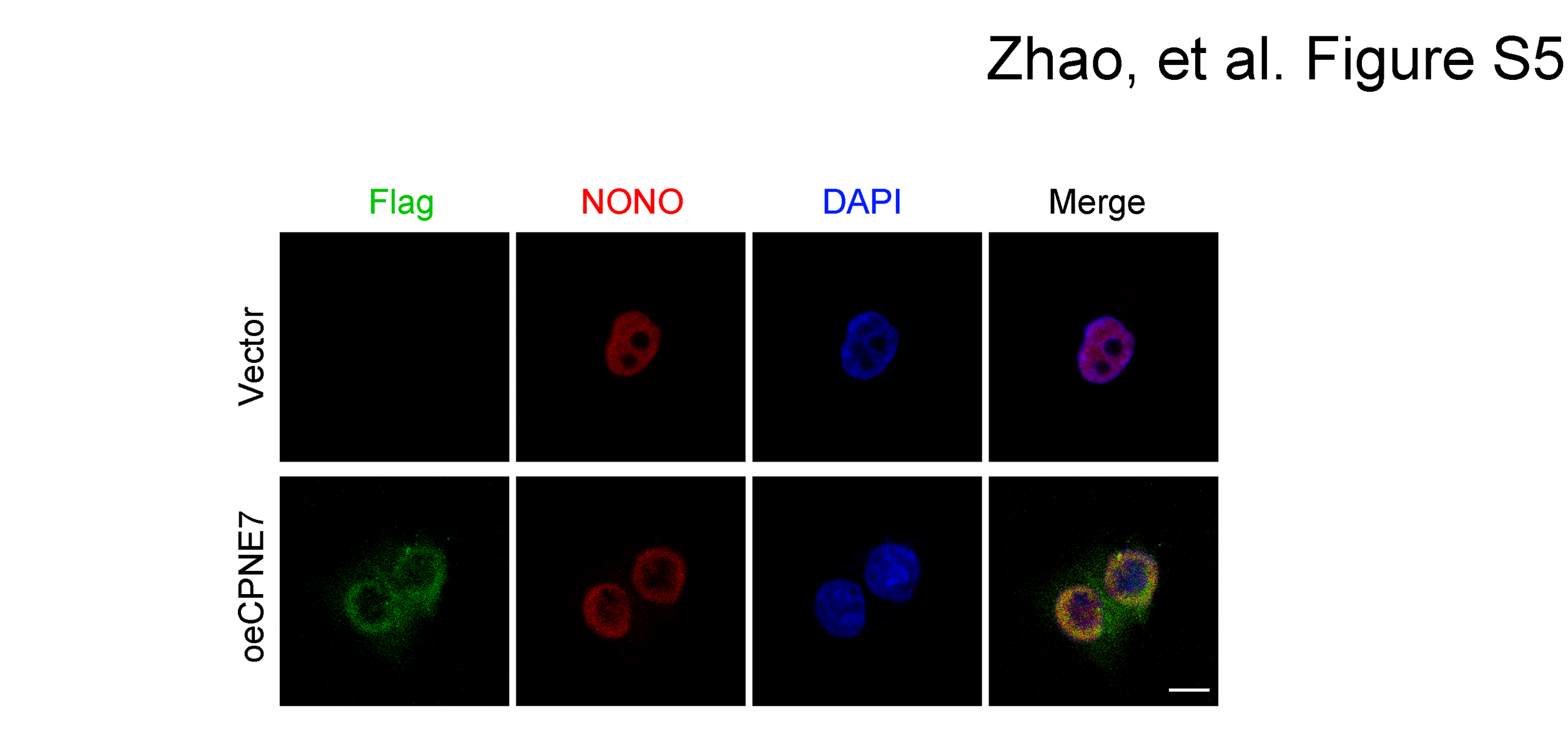


**Supplementary Figure 5. Exogenous CPNE7 colocalizes with NONO in the nucleus.** Immunofluorescence staining of CPNE7 and NONO in CPNE7-Flag overexpressing HCT116 cells. Scale bar: 10 μm.

**Supplementary Table 1. Sequences for qRT-PCR**

| Name | Primer sequence (5’-3’) |
| --- | --- |
| CPNE7-F | CTGGTCTGGGATTACGACTCT |
| CPNE7-R | CAGCCGCCCATGATATAGTCC |
| ACTB-F | CATGTACGTTGCTATCCAGGC |
| ACTB-R | CTCCTTAATGTCACGCACGAT |
| ZFP42-F | TAGAATGCGTCATAAGGGGTGA |
| ZFP42-R | TCTTGCCTGTCATGTACTCAGAA |
| NONO-F | CTTCCCTGATGCGAGAGAGC |
| NONO-R | ATCCGGCATCATAGTGGCAG |

**Supplementary Table 2. Sequences for shRNAs**

| Name | Primer sequence (5’-3’) |
| --- | --- |
| shCPNE7-1-F | TGGAGAATTCTCTACCACCTTTCAAGAGAAGGTGGTAGAGAATTCTCCTTTTTTC |
| shCPNE7-1-R | TCGAGAAAAAAGGAGAATTCTCTACCACCTTCTCTTGAAAGGTGGTAGAGAATTCTCCA |
| shCPNE7-2-F | TGCAGAAGAGACGCAGTTATTTCAAGAGAATAACTGCGTCTCTTCTGCTTTTTTC |
| shCPNE7-2-R | TCGAGAAAAAAGCAGAAGAGACGCAGTTATTCTCTTGAAATAACTGCGTCTCTTCTGCA |
| shZFP42-F | TGGCCTTATGTGATGGCTATTTCAAGAGAATAGCCATCACATAAGGCCTTTTTTC |
| shZFP42-R | TCGAGAAAAAAGGCCTTATGTGATGGCTATTCTCTTGAAATAGCCATCACATAAGGCCA |

**Supplementary Table 3. Sequences for sgRNAs**

| Name | Primer sequence (5’-3’) |
| --- | --- |
| sgCPNE7-1-F | CACCGCTCGGGGAACAACGGCTACG |
| sgCPNE7-1-R | AAACCGTAGCCGTTGTTCCCCGAGC |
| sgCPNE7-2-F | CACCGTTGAGGTGTACGACACGCAT |
| sgCPNE7-2-R | AAACATGCGTGTCGTACACCTCAAC |

**Supplementary Table 4. Sequences for overexpression**

| Name | Primer sequence (5’-3’) |
| --- | --- |
| oeCPNE7-F | ATTTCCGGTGAATTCCTCGAGATGAGCGCGGGCTCGGAG |
| oeCPNE7-R1 | TCACTTGTCATCGTCGTCCTTGTAATCCGGTGTGCAGCCTGGGCT |
| oeCPNE7-R2 | CCGCTCTAGAACTAGTCTCGAGTCACTTGTCATCGTCGTCCTTGTAATC |
| oeNONO-F | TAGTCCAGTGTGGTGGAATTCGCCACCATGGGGATGCAGAGTAATAAAACTTTTAACTT |
| oeNONO-R | TGCTGGATATCTGCAGAATTCTTGTATCGGCGACGTTTGTTT |

**Supplementary Table 5. Sequence of ZFP42 promoter**

| Name | Sequence (5’-3’) |
| --- | --- |
| -2000~  -1700 | ttaagggagtgataaagggtggtttccgacaacccaaagaaaggatcctcggcttgagctaagtgtgtgtgtttccatctagtgaggaaaggggctttcaatgacattgccacttaaaggcttacaaagaaggttctggtcaacattcttcggttcattgaagtatttcttatcgaaacccaaatgttaacatttttatctccaagggaaacaaaaaaaaaggctggtggaattcccctgtagtttctaaagaggccacagcaaggcgccatagagacagaaggagggaagttgagggaa |
| -200~  +100 | gcgccgcccaggtgccaggcggctccgggcagagagtgaacgcgcgggccaggtggctccagggcggcgccccagggcggggccggccacgccctccctaaccctggcggagctgatgggtggctgtagcctgattagaccgcgtcagtccggagggtgggtcttgggagggggcgcagggcagtccacgtttccactgcagtttctcctttgttttacgtttgggaggaggtggcattggaaatagcagagtgcttcgcggtaacaggggtgagtcttgtttcatggaacttttttcaa |
| -2000~  +100 | ttaagggagtgataaagggtggtttccgacaacccaaagaaaggatcctcggcttgagctaagtgtgtgtgtttccatctagtgaggaaaggggctttcaatgacattgccacttaaaggcttacaaagaaggttctggtcaacattcttcggttcattgaagtatttcttatcgaaacccaaatgttaacatttttatctccaagggaaacaaaaaaaaaggctggtggaattcccctgtagtttctaaagaggccacagcaaggcgccatagagacagaaggagggaagttgagggaagtccttctgactggtaaatcggctttctgaccctcgtctgcaaataatacattctaaggtaaggaactaaggaagctttattgaaatcggatttcaaatggagaggtcctgctatgcctactggaggagatcagggaataataaatatttccttcattgaaaattatatcctattggccaggcgcagtggctcacgcctgtaatcccaacactttgggaggccgagacaggcggatcacctgaggtcggaccagcctgccaacatggagaaaccctgtctctactaaaaatactaaattagccgtgcgtggtggtgcatgcctgtaatcccagctactggggaggctgaggcaggagaatcacttgaaccaaataggcggaggttgcagtgaggtgagacggtgccactgcactccagcctgggggacagagaaagactccgtctcaaaaaagaaaaaaaaagagaaagaaaagaaaggaaggaaggaaggaaggaaggaaggaaggaaggaaggaaggaaggaaggaaggaaaattatattttattggccaacctcaaaattgaagcttattcagaaacagatttaaaaaaagaaaaaagtggagaactgctcccagatatatatgcagaaaagcttcaagtcaacatttaggaggtttttaaatatcgatctgggtttcgttctaatggtctgggtgtacttatacgacctattacatttcactagactgtagccaatagtgagcgttgactgaccattgctttcaaagtgagttgtgttgcctttagcaatacagtcacattaatggccagaaaaagaaaaaagaaaacaaacactgggggtgtttgaaacaactgggggtagggtggggtgggaaaggtgtgggggtttgcgttcgagtcaacctctatttcggggggtaaaaactgcctcctccgcttgatttttttcctcgatctgcaaaagcgtaaatgttttgttttgttctattgagatggagcctcgctctatcacccaggctggagcttagtggcgcgatctcggctcactgcaacctccgcatcccgggttcaagcgattctcctgcctcagcctcctgagtagctgggattacaggcgagcaccaccatgcctggctaatttttgtatttttcggtagagacggggtttcgccatgttggccaggctggtctcgaacgcttgacctcatgtgatccccccgtctcggcctcccaaagtgctgggacgacaggcgtgagccaccgcgtccggcctaaaagggtaaatgtgattacacccacgcgtatttgttcaacagacatttattgagcgctcaccacgtgccaacgccgggcgtctgggctctggaggcgctgccacgtggcggatgcgcagtgcccggcggccgggctgaggggtgaacgcgggggtcagggggccccgggctgagggtttgcgtgcgggccgggtgcctgggggccccgggctgagggtgagcgcgccgcccaggtgccaggcggctccgggcagagagtgaacgcgcgggccaggtggctccagggcggcgccccagggcggggccggccacgccctccctaaccctggcggagctgatgggtggctgtagcctgattagaccgcgtcagtccggagggtgggtcttgggagggggcgcagggcagtccacgtttccactgcagtttctcctttgttttacgtttgggaggaggtggcattggaaatagcagagtgcttcgcggtaacaggggtgagtcttgtttcatggaacttttttcaa |
