# Supplementary figures and images for: CPNE7 promotes colorectal tumorigenesis by interacting with NONO to initiate ZFP42 transcription
Source: Cell Death Dis. 2024 Dec 18;15(12):896. doi: 10.1038/s41419-024-07288-z (PMC11655532; doi:10.1038/s41419-024-07288-z)

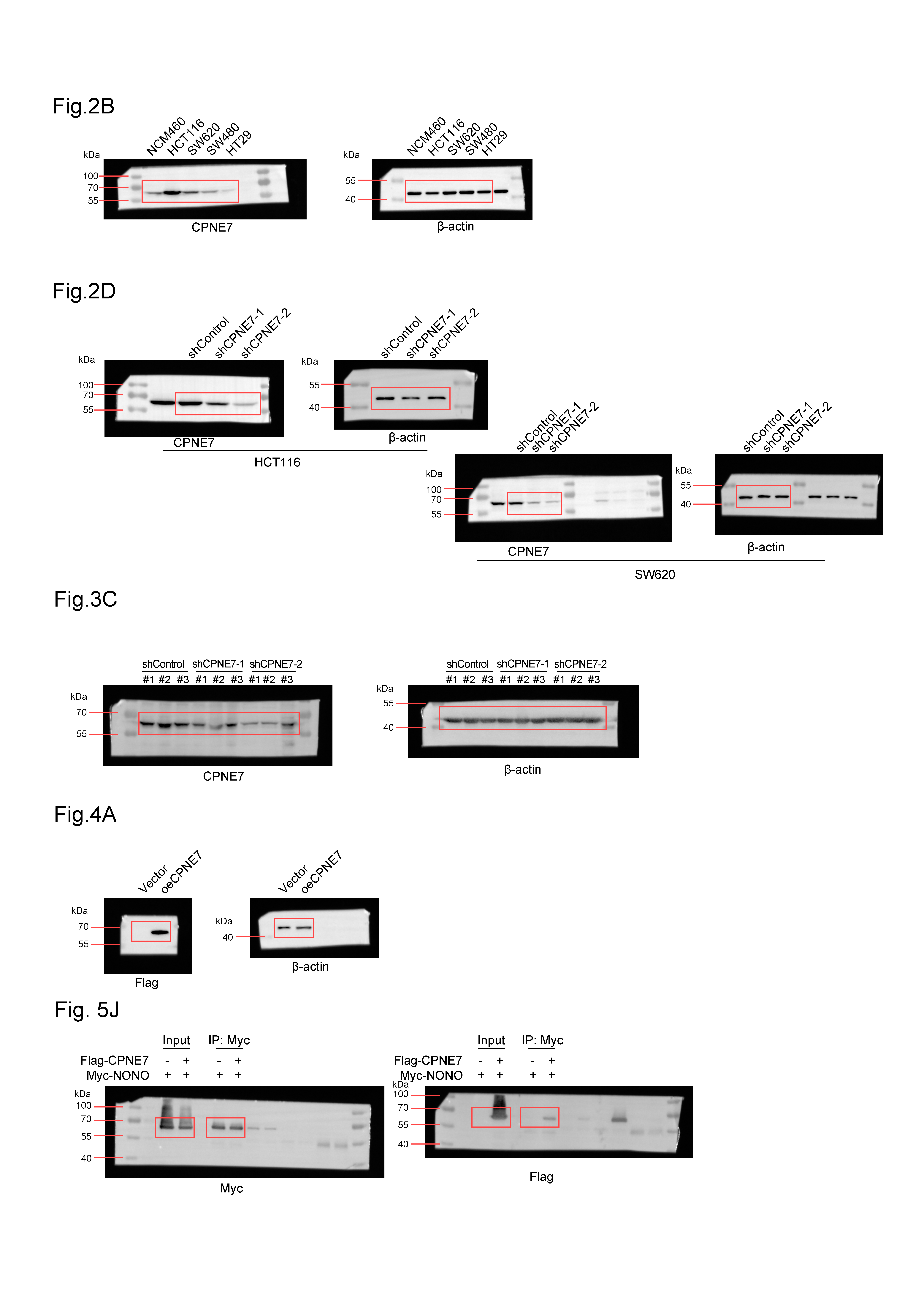

Supplement: Supplementary file 2 — Uncropped Western blots-1 [file 41419_2024_7288_MOESM2_ESM.tif]

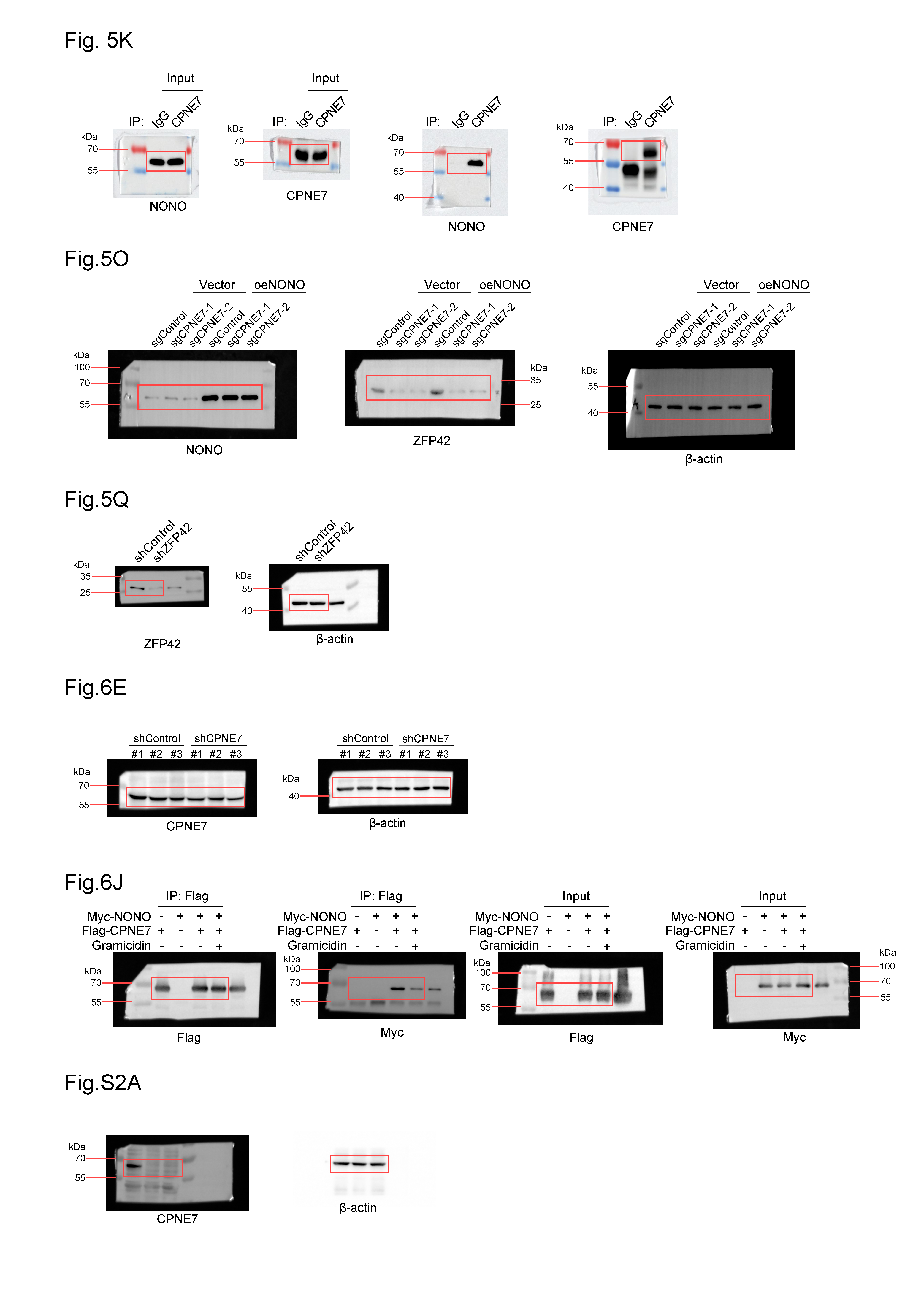

Supplement: Supplementary file 3 — Uncropped Western blots-2 [file 41419_2024_7288_MOESM3_ESM.tif]
